# Hepatic steatosis in HIV-HCV coinfected patients receiving antiretroviral therapy is associated with HCV-related factors but not antiretrovirals

**DOI:** 10.1186/1756-0500-5-180

**Published:** 2012-07-09

**Authors:** Valrie Martinez, Thi Dieu Ngan TA, Zahra Mokhtari, Marguerite Guiguet, Patrick Miailhes, Marc-Antoine Valantin, Frderic Charlotte, Philippe Bertheau, Jean-Michel Molina, Christine Katlama, Eric Caumes

**Affiliations:** 1 Service de Mdecine Interne et Immunologie Clinique, Assistance Publique-Hpitaux de Paris, INSERM UMR_S 996, Universit Paris Sud, Hpital Antoine Bclre, 157, rue de la Porte de Trivaux, 92141, Clamart, France; 2 Service des Maladies Infectieuses et Tropicales, Hpital Piti-Salptrire, Universit Pierre et Marie Curie, APHP, 45/83 Boulevard de lHpital, 75013, Paris, France; 3 Departement of Infectious Disease, Hanoi Medical University, 01, Ton That Tung Street, Hanoi, Vietnam; 4 Departement of Internal Medicine, Hospital National Iranian oil company, Hafez Avenue, Tehran, Iran; 5 INSERM U943, UPMC Univ Paris 06, UMR S943, Paris, F-75013, France; 6 Service de Maladies Infectieuses et Tropicales, 103 Grande-Rue de la Croix-Rousse, Hpital de la Croix-Rousse, Hospices Civils de Lyon, 69317 Lyon cedex 04, Paris, France; 7 Service dAnatomopathologie, Hpital Piti-Salptrire, Universit Pierre et Marie Curie, APHP, 45/83 Boulevard de lHpital, 75013, Paris, France; 8 Service dAnatomopathologie, Hpital Saint-Louis, Universit Denis Diderot, APHP, 1, avenue Claude Vellefaux, 75010, Paris, France; 9 Service des Maladies Infectieuses et Tropicales, Hpital Saint-Louis, Universit Denis Diderot, APHP, 1, avenue Claude Vellefaux, 75010, Paris, France

**Keywords:** HIV, HCV, steatosis, antiretroviral drugs, genotype 3

## Abstract

**Background:**

In HIV and hepatitis C virus (HCV) coinfected patients, the role of antiretroviral therapy (ART) on hepatic steatosis (HS) remains controversial.

**Methods:**

HIV/HCV coinfected patients receiving ART and previously untreated for HCV who underwent a liver biopsy were included. Cumulative duration of exposure to each antiretroviral was recorded up to liver biopsy date. Logistic regression analyses evaluated factors associated with steatosis and its severity.

**Results:**

184 patients were included: median age 41years, 84% male, 89% Caucasian, 61% with a past history of intravenous drug use. HCV genotypes were 1 (55%), 2 (6%), 3 (26%), and 4 (13%). Median HCV-RNA was 6.18 log_10_ IU/ml. HIV-RNA was undetectable (<400 copies/ml) in 67% of patients. Median CD4 count was 321/mm^3^. All patients had been exposed to nucleoside reverse transcriptase inhibitors (median cumulative exposure 56months); 126 received protease inhibitors (23months), and 79 non-nucleoside reverse transcriptase inhibitors (16months). HS was observed in 102 patients (55%): 41% grade 1; 5% grade 2, and 9% grade 3. In multivariate analysis, HCV genotype 3 and HCV viral load were moderately associated with mild steatosis but strongly with grade 2-3 steatosis. After adjustment for the period of biopsy, no association was detected between HS and exposure to any antiretroviral class or drug, or duration of ART globally or comparing genotype 3 to others.

**Conclusions:**

Among our ART-treated HIV-HCV cohort predominantly infected with genotype 1, 55% of patients had HS which was associated with HCV-related factors, but not ART class or duration of exposure.

## Introduction

As a result of shared transmission routes, hepatitis C virus (HCV) infection is common in patients infected with human immunodeficiency virus (HIV) [1-3]. In the USA and Western Europe, at least 30% of HIV-infected patients are also infected with HCV [1-4]. The immunosuppression induced by HIV accelerates the natural history of HCV-related liver disease and the progression of chronic hepatitis C to cirrhosis and end-stage hepatic disease [5-10].

The introduction of highly active antiretroviral therapy has been associated with a dramatic decline in the morbidity and mortality related to specific HIV complications, whereas that related to liver disease has increased significantly in coinfected patients [1,6,11-13]. The relative increase in morbidity and mortality due to liver disease in the HIV population is a composite of accelerated liver disease progression in HCV patients and extended survival of these individuals due to the benefit of antiretroviral therapy (ART).

Hepatic steatosis (HS), defined by the accumulation of lipid droplets in hepatocytes, is present in 24-75% of HIV and HCV coinfected patients [12,14-23]. Some factors contributing to the development of HS in the general population, such as visceral obesity, alcohol consumption, hypertriglyceridemia, hypertension and diabetes mellitus remain hugely discrepant during coinfection [24-28]. In HIV and HCV coinfected patients, HS may occur as a result of the HIV infection or as a consequence of concomitant HCV infection, as well as metabolic factors such diabetes, obesity or antiretroviral drugs which could induce metabolic syndrome, lipodystrophy or lactic acidosis due to mitochondrial damage [12,14,15,18-20,22,29-32]. Nevertheless, the role of ART, particularly stavudine exposure, remains controversial. Moreover, in some studies, HS appears to be more common and severe in coinfected than in HCV-monoinfected patients [16,22] and influence by the viral genotype [16,22]. Borghi et al., showed previously that HIV related steatosis increase in genotype 3 patients and a putative role of ART in patients infected by HCV genotype other than 3 [23]. Other factors besides immunesuppression account for faster progression to ESLD in coinfected patients (i.e. HIV hepatocyte infection, drug liver toxicity).

To assess the prevalence and risk factors of HS, particularly characteristics associated with severity, we reviewed the epidemiological, clinical and biological data of HIV-HCV coinfected patients receiving ART, before HCV therapy and at the time of liver biopsy. Moreover, we compared the effect of ART according to the genotype.

## Patients and Methods

### Patients

For this study, HIV-HCV coinfected patients were retrospectively screened in histopathology databases of two hospitals. All HIV-infected patients with detectable HCV RNA load (qualitative or quantitative detection), receiving ART but naive of HCV-specific therapy and who underwent a liver biopsy between January 1995 and January 2008, were included. When repeated liver biopsies were performed, only data associated with the first one was studied.

Patients were excluded if they were positive for hepatitis B surface antigen, had a negative plasma HCV RNA load, or other chronic liver diseases, such as autoimmune hepatitis, hemochromatosis, Wilsons disease or alpha-1 antitrypsin deficiency. All patients have been tested for all of these parameters and the patients included were negative.

At the day of liver biopsy, the following variables were assessed: age, gender, ethnicity, alcohol (reported by physician in the medical report, but no data about the quantity consumed daily), intravenous drug abuse, duration of documented HIV and HCV infections, risk factors for viral transmission, CD4 cell count, HIV and HCV plasma levels, HCV genotype, fasting glycemia, total cholesterolemia and triglyceridemia, alanine aminotransferase (ALT), aspartate aminotransferase (AST), gammaglutamyl transferase (GGT) and alkaline phosphatase. Measure of insulinemia, not performed in clinical practice, was not available in this retrospective study, nor measure of weight, body mass index and waist circumference which were not recorded in the medical report. Metabolic parameters were also recorded for each patient on a fasting state. For the purposes of this study, metabolic syndrome was defined as triglycerides >1.7mmol/l and glycemia 5.6mmol/l as recommended by the International Diabetes Federation 2005 and diabetes as a fasting glycemia>7mmol/l and excluded waist circumference [33].

HCV RNA detection was performed using a signal amplification nucleic acid probe assay (bDNA 3.0, Bayer diagnostics, Tarrytown NY) and was expressed in KIU/ml. All biological data were assessed directly to the data system of the different laboratories of the 2 hospitals. All of the parameters collected were routinely performed in HIV-HCV patients who underwent a liver biopsy and were assessed on the day of biopsy or during the week before.

History of antiretroviral therapy was assessed in the medical report and from our database (Nadis software). The cumulative duration of exposure to each drug and class of drugs was recorded for each patient up to the date of liver biopsy. As patients could have received more than one ART regimen, and because of the large diversity of regimens available in France, it would be impossible to select specific drug combinations for analysis. Therefore, we hypothesized that a relationship between a combination of drugs and severity of HS would be evident by studying each component of the combination.

### Histologic evaluation

Percutaneous or transjugular liver biopsy specimens were fixed in formalin and embedded in paraffin. Minimal size was 10mm and contained at least 6 portal spaces, excepted if cirrhosis. Sections 4m thick were stained with hematoxylin and eosin, with picrosirius stain for collagen and Perls stain for iron. All liver biopsy specimens were evaluated by two experienced pathologists, one in each hospital (F.C. or P.B.).

The grade of activity and the stage of fibrosis were evaluated according to the METAVIR scale [34]. Necroinflammatory activity was graded A0 (none), A1 (mild), A2 (moderate) or A3 (high). The degree of portal and septal fibrosis was assessed as F0 (none), F1 (portal fibrosis without septa), F2 (portal fibrosis with a few septa), F3 (portal fibrosis with numerous septa) or F4 (cirrhosis). HS was evaluated and graded as proposed by Brunt et *al.*[33]: grade 0, none; grade 1, steatosis involving <33% of hepatocytes; grade 2, 33-66% and grade 3, >66%. HS was defined as mild (grade 1) or severe (grade 2-3).

### Statistical analysis

Median and interquartile ranges (IQRs) described continuous variables. Comparisons between patients found with and without steatosis were performed using Kruskal-Wallis or Wilcoxon tests for quantitative variables, and ^2^ test or Fishers exact test for qualitative variables. Logistic regression analyses were used to identify determinants of liver steatosis, and polytomous logistic regression analyses evaluated factors associated with the presence of mild or severe steatosis.

Exposure to ART was studied after adjustment for the period of biopsy (1995-1998, 1999-2001, 2002-2008). The periods were chosen at the time of the statistical analysis according to the introduction of drugs such as abacavir in 1999 and tenofovir in 2002 to show an impact of the use of more metabolic friendly drugs. Exposure to ART and cumulative exposure duration (per 1year increased) were evaluated globally for all patients and according to genotype: genotype 3 compared to others.

Variables with *p*<0.15 in univariate analyses were included in the final model. Analyses were processed with the use of SAS software (SAS Institute, Cary, North Carolina, USA).

## Results

### Study population

Between January 1995 and January 2008, 250 HIV-HCV coinfected patients who underwent a liver biopsy and fulfilled all inclusion criteria were evaluated. Sixty-six patients were not analyzed for the following reasons corresponding to exclusion criteria: hepatitis B coinfection (n=28), loss of medical records (n=8) or no ART at the time of biopsy (n=30). Therefore, 184 patients were included. Demographic and biological characteristics are summarized in Table1.

**Table 1:** Characteristics of HIV-HCV coinfected pati**e**nts treated with antiretroviral therapy at the time of liver biopsy (parameters collected the day of the liver biopsy or during the week before)

	n=184
Median age at biopsy (IQR), years	41 (36-45)
Male, n (%)	154 (84)
Ethnicity, n (%)	
Caucasian	163 (89)
Black	21 (11)
Intravenous drug use, n (%)	127 (61)
Alcohol use, n (%)*	67 (36)
Median duration of HIV diagnosis (IQR, years	11 (7-14)
Median CD4 cell counts/mm^3^ (IQR)	321 (227-461)
Undetectable HIV RNA^+^, n (%)	119 (67)
HCV genotype^++^, n (%)	
1	98 (55)
2	10 (6)
3	47 (26)
4	24 (13)
Median HCV RNA (IQR), log_10_ KIU/ml^+++^	6.18 (5.76-6.60)
Median laboratory variables (IQR), U/l	
ALT	83 (47-128)
AST	65 (43-97)
Alkaline phosphatase	89 (71-113)
GGT	80 (42-160)
Median glycemia (IQR), mmol/l	4.8 (4.3-5.3)
Glycemia 5.6mmol/l^+^, n (%)	33 (19)
Glycemia7.0mmol/l^+^, n (%)	7 (4)
Median total cholesterol (IQR), mmol/l	4.20 (3.54-5.10)
Median triglyceridemia (IQR), mmol/l	1.49 (0.99-2.10)
Triglyceridemia 1.7mmol/l^+^, n (%)	60 (36)
Median duration of antiretroviral therapy (IQR), months	58 (28-94)
Median cumulative exposure to antiretrovirals (IQR), months	
NRTI	56 (28-90)
NNRTI	16 (9-31)
PI	23 (12-40)
Combination of ART at liver biopsy, n (%)	
2 NRTI+1 PI	93 (51)
2 NRTI+1 NNRTI	36 (20)
1 or 2 NRTI	31 (16)
3 NRTI	17 (9)
Other regimens	7 (4)

^+^ proportions calculated on available data.

^+^ Data missing for 6 patients; ^++^ Data missing for 5 patients; ^+++^ Data missing for 36 patients; Data missing for 11 patients; Data missing for 18 patients.

*Alcohol: no quantification was available.

*Abbreviations:* ALT, alanine transaminase; AST, aspartate aminotransferase; GGT, gamma-glutamyl transpeptidase; IQR, interquartile range; NNRTI, non-nucleoside reverse transcriptase inhibitor; NRTI, nucleoside reverse transcriptase inhibitor; PI, protease inhibitor.

No correlation was observed between the duration of HIV infection and CD4 cell count in this cohort of ART-treated patients. HIV plasma viral load was undetectable (<400 copies/ml) in 119 out of the 178 (67%) evaluable patients and median HIV viral load on average was 3.93 log_10_ copies/ml for the remaining 33% patients. The median HCV viral load was 6.18 log_10_ IU/ml (IQR 5.76-6.60) in the 148 (80%) patients with quantificative HCV values. The other patients (n=36) had only a positive HCV RNA without quantification.

All patients had been exposed to a nucleoside reverse transcriptase inhibitor (NRTI), including zidovudine (n=144), lamivudine (n=161), stavudine (n=114), zalcitabine (n=22), didanosine (n=105), tenofovir (n=24) and abacavir (n=24); 126 patients had received protease inhibitors (PI), including ritonavir as boosted-PI (n=59), indinavir (n=68), nelfinavir (n=47), saquinavir (n=16), lopinavir (n=17) and atazanavir (n=8); and 79 patients had received non-nucleoside reverse transcriptase inhibitors (NNRTI), including efavirenz (n=51) and nevirapine (n=44).

### Histologic findings

Overall, 102 (55%) out of the 184 patients had HS. Steatosis was grade 1 in 76 (41%) patients, grade 2 in 10 (5%), and 3 in 16 (9%). Macrovesicular fatty changes were observed in 56 patients (55%), microvesicular in 10 (10%), and mixed form in 36 (35%). Fibrosis was present in 163 patients (89%) with METAVIR F1 score in 66 (36%), F2 in 53 (29%), F3 in 39 (21%) and F4 in 4 (2%). Necroinflammatory activity was detected in 164 out of the 184 (89%) patients with A1 in 109 (59%), A2 in 50 (27%) and A3 in 5 (3%).

### Factors associated with hepatic steatosis

Comparison of parameters in patients with or without HS are presented in Table2. In univariate analysis, intravenous drug use (*p*=0.05) and HCV genotype 3 (*p*=0.005) were strongly linked with HS. The median HCV viral load was significantly higher in patients with HS compared with those without (*p*=0.003). HS was also associated with increased levels of ALT, AST and decreased levels of serum total cholesterol, but not with triglyceridemia nor available parameters of metabolic syndrome (only 7 patients have a glycemia >7mmol/l with mild steatosis for 6 patients and 1 had no steatosis) (Table2).

**Table 2:** Comparison of various parameters in patients with mild (<33% of hepatocytes affected) or severe (>33% of hepatocytes affected) steatosis and those without steatosis (univariate analysis)

	No steatosis (n=82)	Steatosis (n=102)	Mild steatosis (n=76)	Severe steatosis (n=26)	*p*^*a*^	*p*^b^	*p*^c^
Median age (IQR), yrs	40 (36-45)	41 (37-45)	41 (36-45)	41 (37-48)	0.38	0.66	0.81
Male gender, n (%)	66 (80)	88 (86)	67 (88)	21 (81)	0.32	0.39	0.34
IVDU, n (%)	50 (62)	77 (76)	54 (71)	23 (88)	0.05	0.03	0.11
Alcohol use, n (%)	29 (35)	38 (37)	29 (38)	9 (35)	0.88	0.92	0.82
Median duration of HIV (IQR), yrs	10 (5-14)	12 (8-14)	12 (8-15)	11 (7-13)	0.17	0.23	0.30
Median CD4 cell count (IQR), /mm^3^	346 (228-560)	308 (227-423)	305 (227-435)	313 (191-398)	0.10	0.24	0.67
HIV RNA<400 copies/ml, n (%)	58 (72)	61 (63)	46 (62)	15 (65)	0.26	0.45	1.0
HCV genotype 3, n (%)	13 (16)	34 (35)	18 (25)	16 (64)	0.005	<.0001	<.0001
Median HCV RNA (IQR), logIU/ml	5.97 (5.65-6.50)	6.30 (6.00-6.72)	6.22 (5.80-6.64)	6.69 (6.27-7.06)	0.003	0.0003	0.004
Median ALT (IQR), U/l	67 (40-119)	87 (54-130)	83 (51-121)	113 (84-132)	0.04	0.01	0.03
Median AST (IQR), U/l	56 (37-78)	77.5 (50-104)	70 (48-102)	78 (59-105)	0.001	0.003	0.33
Median alkaline phosphase (IQR), U/l	90 (70-115)	89 (69-112)	90 (72-113)	80 (65-95)	0.46	0.27	0.15
Median GGT (IQR), U/l	71 (41-139)	87 (43-196)	92 (44-198)	73 (30-180)	0.09	0.09	0.19
Median glycemia (IQR), mmol/l	4.80 (4.30-5.45)	4.92 (4.30-5.22)	4.80 (4.30-5.20)	4.90 (4.35-5.26)	0.79	0.96	0.97
Glycemia 5.6mmol/l, n (%)	16 (21)	17 (17)	12 (16)	5 (21)	0.56	0.77	0.76
Glycemia 7.0mmol/l, n (%)	1 (1)	6 (6)	6 (8)	0 (0)	0.14	0.08	0.33
Median total cholesterol (IQR), mmol/l	4.40 (3.73-5.33)	4.00 (3.30-4.79)	4.01 (3.30-5.03)	3.72 (3.06-4.37)	0.01	0.02	0.30
Median triglycerides (IQR), mmol/l	1.47 (0.97-2.08)	1.51 (0.99-2.20)	1.53 (1.04-2.24)	1.23 (0.90-1.74)	0.86	0.51	0.28
Triglycerides 1.7mmol/l, n (%)	30 (40)	30 (33)	23 (33)	7 (29)	0.33	0.56	0.80
Fibrosis F3-F4, n (%)	12 (15)	31 (30)	25 (33)	6 (23)	0.01	<0.001	1.0
Metavir score activity A2/A3, n (%)	21 (26)	34 (33 )	29 (38 )	5 (19 )	0.28	0.11	0.09

p^a^ Comparison of patients with versus without steatosis (Kruskal-Wallis test for quantitative variables, ^2^ test or Fisher's exact test for qualitative variables).

p^b^ Comparison of patients with mild or severe steatosis versus without steatosis (Kruskal-Wallis test for quantitative variables, ^2^ test for qualitative variables).

p^c^ Comparison of patients with mild versus severe steatosis (Kruskal-Wallis/Wilcoxon test for quantitative variables, ^2^ test or Fisher's exact test for qualitative variables).

Also, there was no association with CD4 cell count or an undetectable HIV viral load. Similar results were found in patients with mild versus severe steatosis.

**Figure 1:**
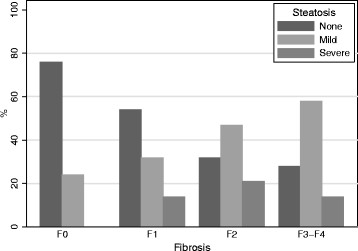
**Distribution of steatosis according to fibrosis index in 184 HIV-HCV coinfected patients treated with antiretroviral therapy.** Steatosis is defined as mild (<33% of hepatocytes affected) or severe (>33% of hepatocytes affected).

HS was also associated with fibrosis (Figure1). Thus, the frequency of HS increased with the stages of fibrosis, with 76%, 54%, 32%, and 28% of patients without steatosis having fibrosis scores of F0, F1, F2, and F3F4, respectively (Cochran-Armitage trend test, *p*<0.0001). However, the severity of steatosis was not different among patients presenting fibrosis scoring F1, F2, or F3F4. No association was observed between HS and necroinflammatory activity.

After adjustment for the period of biopsy, neither the type of ART nor the duration of exposure to a specific antiretroviral agent or class of antiretroviral was related to HS (Table3). The same results were observed in separate analyses among patients presenting genotype 3, or another genotype (Tables4 and 5).

**Table 3:** The effects on steatosis of exposure to specific antiretroviral medication in 184 HIV-HCV coinfected patients

Antiretrovirals	n	Exposure to ARV Adjusted OR (95% CI)	*p*	Cumulative exposure duration (per 1year increase) Adjusted OR (95% CI)	*p*
Nucleoside reverse transcriptase inhibitors	184	NE		1.05 (0.961.16)	0.30
zidovudine	144	1.11 (0.54-2.30)	0.77	1.03 (0.91-1.16)	0.65
lamivudine	161	1.10 (0.44-2.77)	0.82	1.01 (0.88-1.17)	0.84
stavudine	114	0.96 (0.51-1.78)	0.88	1.05 (0.90-1.24)	0.54
didanosine	105	1.20 (0.66-2.18)	0.57	1.04 (0.85-1.27)	0.70
zalcitabine	22	1.68 (0.60-4.72)	0.33	1.50 (0.68-3.34)	0.31
tenofovir	24	0.87 (0.33-2.27)	0.78	1.19 (0.69-2.07)	0.53
abacavir	24	0.55 (0.22-1.39)	0.20	0.98 (0.69-1.41)	0.94
Protease inhibitors	126	1.15 (0.59-2.24)	0.67	1.06 (0.90-1.25)	0.50
ritonavir	55	0.90 (0.46-1.75)	0.75	0.96 (0.71-1.28)	0.76
indinavir	68	0.94 (0.51-1.74)	0.84	1.14 (0.87-1.47)	0.34
nelfinavir	47	0.96 (0.48-1.89)	0.91	1.12 (0.79-1.59)	0.54
saquinavir	31	0.91 (0.41-2.00)	0.81	0.72 (0.45-1.14)	0.16
lopinavir-ritonavir	17	0.55 (0.19-1.63)	0.28	0.86 (0.54-1.37)	0.52
atazanavir	8	0.76 (0.17-3.43)	0.72	0.99 (0.35-2.81)	0.98
Non-nucleoside reverse transcriptase inhibitors	79	0.93 (0.50-1.74)	0.81	1.17 (0.88-1.55)	0.27
efavirenz	51	1.34 (0.67-2.69)	0.40	1.19 (0.88-1.63)	0.26
nevirapine	44	0.67 (0.33-1.35)	0.26	1.24 (0.77-2.00)	0.36

Adjusted for period of liver biopsy.

Abbreviations: OR, odds ratio; CI, confidence intervals; NE, not estimable.

**Table 4:** The effects on steatosis of exposure to specific antiretroviral medication in 184 HIV-HCV coinfected patients according to the genotype

		All patients (n=184)		Genotype 3 (n=47)		Other genotype (n=132)	
Antiretrovirals	n	Adjusted OR (95% CI)	*p*	Adjusted OR (95% CI)	*p*	Adjusted OR (95% CI)	*p*
NRTI	184	NE		NE		NE	
zidovudine	144	1.11 (0.542.30)	0.77	0.91 (0.22-3.82)	0.90	1.42 (0.58-3.49)	0.45
lamivudine	161	1.10 (0.442.77)	0.82	0.50 (0.05-5.00)	0.56	1.29 (0.45-3.74)	0.64
stavudine	114	0.96 (0.511.78)	0.88	0.58 (0.14-0.47)	0.46	1.05 (0.51-2.17)	0.89
didanosine	105	1.20 (0.662.18)	0.57	1.02 (0.27-3.93)	0.97	1.17 (0.58-2.37)	0.66
zalcitabine	22	1.68 (0.604.72)	0.33	NE		1.29 (0.41-4.06)	0.66
tenofovir	24	0.87 (0.332.27)	0.78	0.15 (0.01-1.80)	0.13	1.44 (0.49-4.22)	0.51
abacavir	24	0.55 (0.221.39)	0.20	0.40 (0.07-2.40)	0.32	0.58 (0.18-1.84)	0.35
PI	126	1.15 (0.592.24)	0.67	0.19 -0.02-1.76)	0.15	1.46 (0.67-3.15)	0.34
ritonavir	55	0.90 (0.461.75)	0.75	0.54 (0.13-2.28)	0.40	1.04 (0.48-2.27)	0.91
indinavir	68	0.94 (0.511.74)	0.84	1.13 (0.29-4.41)	0.86	0.81 (0.39-1.68)	0.57
nelfinavir	47	0.96 (0.481.89)	0.91	0.37 (0.09-1.56)	0.18	1.19 (0.54-2.66)	0.66
saquinavir	31	0.91 (0.412.00)	0.81	0.41 (0.10-1.72)	0.22	0.94 (0.34-2.61)	0.91
lopinavir-ritonavir	17	0.55 (0.191.63)	0.28	0.45 (0.02-8.11)	0.59	0.67 (0.20-2.19)	0.50
atazanavir	8	0.76 (0.173.43)	0.72	NE		1.70 (0.31-9.24)	0.54
NNRTI	79	0.93 (0.501.74)	0.81	0.33 (0.08-1.27)	0.11	1.38 (0.65-2.95)	0.40
efavirenz	51	1.34 (0.672.69)	0.40	0.74 (0.17-3.27)	0.69	1.77 (0.79-4.00)	0.16
nevirapine	44	0.67 (0.331.35)	0.26	0.34 (0.08-1.38)	0.13	0.80 (0.35-1.83)	0.60

Adjusted for period of liver biopsy.

Abbreviations: OR, odds ratio; CI, confidence intervals; NE, not estimable.

**Table 5:** The effects on steatosis of cumulative exposure duration to specific antiretroviral medication in 184 HIV-HCV coinfected patients according to the genotype

	All patients (n=184)		Genotype 3 (n=47)		Other genotype (n=132)	
Antiretrovirals	Cumulative exposure duration (per 1year increase) Adjusted OR (95% CI)	*p*	Cumulative exposure duration (per 1year increase) Adjusted OR (95% CI)	*p*	Cumulative exposure duration (per 1year increase) Adjusted OR (95% CI)	*p*
Nucleoside reverse transcriptase inhibitors	1.05 (0.96-1.16)	0.30	1.07 (0.85-1.35)	0.55	1.03 (0.92-1.15)	0.64
zidovudine	1.03 (0.91-1.16)	0.65	1.01 (0.81-1.27)	0.92	1.00 (0.86-1.17)	0.98
lamivudine	1.01 (0.88-1.17)	0.84	0.99 (0.75-1.32)	0.97	0.99 (0.84-1.17)	0.92
stavudine	1.05 (0.90-1.24)	0.54	1.16 (0.81-1.68)	0.41	0.99 (0.81-1.22)	0.94
didanosine	1.04 (0.85-1.27)	0.70	1.08 (0.64-1.81)	0.77	1.04 (0.83-1.29)	0.75
zalcitabine	1.50 (0.68-3.34)	0.31	NE		1.35 (0.57-3.16)	0.49
tenofovir	1.19 (0.69-2.07)	0.53	0.29 (0.03-2.49)	0.26	1.46 (0.80-2.66)	0.22
abacavir	0.98 (0.69-1.41)	0.94	1.14 (0.54-2.41)	0.73	0.88 (0.56-1.41)	0.61
Protease inhibitors	1.06 (0.90-1.25)	0.50	1.14 (0.82-1.59)	0.44	0.98 (0.80-1.21)	0.87
ritonavir	0.96 (0.71-1.28)	0.76	0.88 (0.45-1.74)	0.72	1.00 (0.71-1.40)	0.98
indinavir	1.14 (0.87-1.47)	0.34	NE		0.89 (0.63-1.25)	0.50
nelfinavir	1.12 (0.79-1.59)	0.54	1.06 (0.63-1.76)	0.83	1.11 (0.66-1.87)	0.68
saquinavir	0.72 (0.45-1.14)	0.16	0.67 (0.33-1.36)	0.26	0.54 (0.25-1.19)	0.13
lopinavir-ritonavir	0.86 (0.54-1.37)	0.52	0.90 (0.13-6.28)	0.92	0.89 (0.54-1.46)	0.64
atazanavir	0.99 (0.35-2.81)	0.98	NE		1.53 (0.43-5.47)	0.51
Non-nucleoside reverse transcriptase inhibitors	1.17 (0.88-1.55)	0.27	1.04 (0.60-1.82)	0.89	1.25 (0.90-1.75)	0.18
efavirenz	1.19 (0.88-1.63)	0.26	1.08 (0.56-2.09)	0.82	1.28 (0.89-1.83)	0.18
nevirapine	1.24 (0.77-2.00)	0.36	0.47 (0.19-1.20)	0.57	1.18 (0.67-2.08)	0.56

Adjusted for period of liver biopsy.

Abbreviations: OR, odds ratio; CI, confidence intervals; NE, not estimable.

In the multivariate analysis, only two independent factors remained associated with an increased risk of HS: HCV genotype 3 (odds ratio [OR], 2.6; 95% confidence intervals [CI], 1.1-6.3), and HCV RNA load (OR, 2.2 per 1 log higher; 95% CI, 1.2-3.8). Using a multivariate polytomous logistic regression model, HCV genotype 3 and HCV RNA load were moderately associated with mild steatosis and strongly associated with severe steatosis (Table6).

**Table 6:** Results from multivariate polytomous logistic regression analyses of factors associated with mild (<33% of hepatocytes affected ) and severe (>33% of hepatocytes affected) steatosis in 184 HIV-HCV coinfected patients treated with antiretroviral therapy

	Mild steatosis AOR (95%CI)	*p*	Severe steatosis AOR (95%CI)	*p*
HCV genotype 3	1.76 (0.72-4.30)	0.22	19.51 (4.49-84.73)	<0.0001
HCV RNA (per 1 log10 increase)	1.80 (1.02-3.16)	0.04	14.86 (3.79-58.22)	0.0001

*Abbreviations:* AOR, adjusted odds ratio; CI, confidence intervals.

## Discussion

Hepatic steatosis has emerged as a major comorbidity in HIV-HCV coinfected patients [13,35]. In this retrospective observational study of 184 HIV-HCV coinfected patients ART-treated but untreated for HCV at the time of liver biopsy, HS was present in about half patients (55%), which is similar to rates of 24-75% found in other studies of HIV-HCV coinfected and HCV monoinfected patients [12,14-23,36]. In multivariate analysis, HS and its severity were only significantly associated with HCV genotype 3 and HCV viral load. Neither the type of ART, nor their prolonged duration of exposure with a median of near five years were related to steatosis. Moreover, ART have no differential effect on occurrence of HS according to the genotype 3 compared to others.

We confirmed previously data of higher HS associated with genotype 3 [23]. Here, the rate of severe HS (14%) was higher in our study compared with 2-9% found in US studies in HIV-HCV coinfected individuals [12], but was similar with rates found in other studies [37,38], in particular with those conducted in France [39,40]. Several reasons may explain these discrepancies. Whereas most of our patients, and those included in the study of Bauerle et *al*. [37], were Caucasian and carrying HCV genotype 3, other studies have included a high proportion of Africo-American patients (47-94%) and patients infected with HCV genotype 1 [38-45]. It is well-known that HCV-infected Black people have a lower prevalence of HS than Caucasian [41,45-47], probably related to lower visceral adipose tissue [16,22].

HS is a frequent histological finding in patients with chronic hepatitis C virus infection, particularly among those infected with genotype 3 strain [24,48,49]. The prevalence of 26% of genotype 3 in our study was similar to the prevalence of 18% reported in the French HepaVIH cohort [50]. It has been postulated that genotype 1 is associated with metabolic steatosis rather than viral steatosis developed through a direct cytopathologic effect observed especially in genotype 3 infected patients [51-56]. When we examined the factors impacting the level of steatosis on ART patients, genotype 3, and high HCV viral load were two independent factors associated with HS in accordance with previous studies [15,17,19,20]. Both factors moderately increased the risk of mild steatosis, but were strongly associated with severe steatosis (grade 2 or 3).

Other factors such as greater age [18], higher body mass index (BMI) [12,15-18,20,22,38-40,42,44][23], hyperglycemia [12], lower cholesterolemia [17], and presence of lipodystrophy [17] have also been found to be independently associated with steatosis in coinfected patients. In our study, patients with metabolic syndrome were not more likely to present HS. However, only seven patients had diabetes, and this limited sample size could have prevent to study this risk factor. Moreover, as expected, exposure to PIs and stavudine, were associated with elevated triglycerides (*p*=0.08 and *p*<0.0001, respectively), but this metabolic abnormality was not a risk factors of HS in our ART-experienced population. According to the lack of data about HOMA scoring, weight and BMI, we probably missed the impact of metabolic steatosis in genotype other than 3 [23,41]. Other limitation of our retrospective study is lack of information about alcohol consumption. Nevertheless, as described by Machado et *al.*, metabolic syndrome and alcohol were not associated to HS. BMI was considered an increasing risk factor but with small magnitude and diabetes as a possible risk factor with no data for HOMA scoring [16,22]. Moreover, the higher percentage of genotype 3 reflect the association between HS and HCV viral parameters.

Many antiretroviral drugs have been associated with hepatic damage [57-59]. It has been suggested that steatosis due to an accumulation of fatty acids in the hepatocytes could be a consequence of mitochondrial dysfunction, secondary to drugs or viruses inducing oxidative stress [60]. The effect of the drug class and drugs within classes on HS in HIV-HCV coinfected patients remain unclear. Previously, some studies reported no significant association between HS and ART as in our study [12,15,16,18,41]. When focusing on the use of stavudine, a medication closely linked with HS and lipodystrophy syndrome resulting from mitochondrial damage, Sulkowski et *al.*[45] found that stavudine exposure was a risk factor for steatosis like in the study of Borghi et al., whereas no such association was found for this drug as well as the D-drug group of antiretrovirals (didanosine, zalcitabine) in other studies [23,38,41,42,61,62]. Recently, in a meta-analysis of the risk factors associated with HS in HIV-HCV patients, Machado et *al.* failed to find any association with antiretrovirals of any class and HS as well as in our study [16,22]. Moreover, Woreta et al., showed in a study including a majority of Black patients (87%) with 94% genotype 1, the lack of association with antiretroviral drugs with a median cumulative drug exposure similar to ours [41]. Despite its relatively small sample size, our study had a statistical power of 80% to detect an increased risk of steatosis of 3 for the antiretroviral drugs used less frequently such as abacavir, tenofovir and lopinavir. We could hypothesize that discrepancies with other studies were linked to the differential prevalence of Caucasion subjects, of metabolic characteristics and frequency of genotype 1 and 3. Borghi et *al*. evocated a putative role of ART in the occurrence of HS in patients infected with genotypes other than 3 [23] but in our study, the impact of ART on HS was not different between genotype 3 and the others.

In conclusion, for our Caucasian cohort predominantly infected with genotype-HCV 1, hepatic steatosis in HIV-HCV coinfected patients receiving antiretroviral therapy is associated with HCV-related factors particularly in genotype 3 patients but not antiretrovirals. Nevertheless, as showed in our study, ART seems play a minor role in HS since the choose and use of more "metabolically friendly" antiretroviral drugs. Overall, we found only viral parameters, HCV genotype 3, and HCV RNA value, which were strongly associated with HS, particularly a severe steatosis. Among HIV-HCV co-infected patients receiving ART and who had never been treated for HCV, neither the type of drugs nor the duration of exposure was related to HS whatever the genotype.

## Abbreviations

HIV = human immunodeficiency virus; HCV = hepatitis C virus; ART = antiretroviral therapy; ALT = alanine aminotransferase; AST = aspartate aminotransferase; GGT = gammaglutamyl transferase; IQR = interquartile range; NRTI = nucleoside reverse transcriptase inhibitor; PI = protease inhibitor; NNRTI = non-nucleoside reverse transcriptase inhibitor; OR = odds ratio; CI = confidence intervals; IVDU = intravenous drug use; AOR = adjusted odds ratio; NE = not estimable.

## Meeting presentation

13^th^ Conference of Retroviruses and Opportunistic Infections, 58 February 2006, Denver, Colorado, USA. Poster number: p-169 and 16^th^ Conference of Retroviruses and Opportunistic Infections, 811 February 2009, Montral, Canada. Poster number: p-853.

## **Competing interests**

The authors declare that they have no competing interests.

## **Authors' contributions**

VM conceived the study, collected data of patients, participated in its design and coordination and drafted the manuscript. TDNT and ZM collected the data and helped to draft the manuscript. MG made the statistical analysis and helped to draft the manuscript. PM and MAV helped to draft the manuscript. FC and PB made the histological analysis of liver biopsy. CK and EC participated in the design of the study and helped to draft the manuscript. All authors read and approved the final manuscript.

## **Financial disclosure**

The authors have **no commercial links or other associations** that might pose a conflict of interest (e.g. pharmaceutical stock ownership, consultancy, advisory board membership, relevant patents, or research funding) relevant to this study.

## Statement naming sources of **financial support** (including grant numbers)

None
